# Supplementary material for: Leveraging molecular structure and bioactivity with chemical language models for de novo drug design
Source: Nat Commun. 2023 Jan 7;14:114. doi: 10.1038/s41467-022-35692-6 (PMC9825622; doi:10.1038/s41467-022-35692-6)
Supplement: Supplementary file 1 — Supplementary Information [file 41467_2022_35692_MOESM1_ESM.pdf]

Supplementary Information

Leveraging molecular structure and bioactivity with chemical language models for de novo drug design

Michael Moret, Irene Pachon Angona, Leandro Cotos, Shen Yan, Kenneth Atz, Cyrill Brunner, Martin Baumgartner, Francesca Grisoni & Gisbert Schneider

Supplementary Tables (page S2)

Supplementary Figures (page S6)

Supplementary Methods (page S16)

Supplementary Tables

Supplementary Table 1 | Validity, uniqueness, and novelty of the molecules sampled with different nucleus parameters at the end of the transfer learning. Each experiment was repeated 10 times. For each repeat, 1000 molecules were sampled. The results are shown for epoch 40 (last epoch of the transfer learning). The mean and standard deviation are shown for the 10 repeats. All percentages are reported with respect to the total number of molecules sampled.

Nucleus	Valid (%)	Unique (%)	Novel (%)
0.60	93.7 ± 0.9	54.7 ± 2.8	54.7 ± 2.8
0.65	93.4 ± 0.9	53.9 ± 3.7	53.9 ± 3.7
0.70	94.1 ± 0.8	53.4 ± 1.9	53.4 ± 1.9
0.75	93.1 ± 0.9	53.9 ± 2.6	53.9 ± 2.6
0.80	93.6 ± 1.2	53.3 ± 4.1	53.3 ± 4.1
0.85	93.7 ± 0.6	54.0 ± 3.4	54.0 ± 3.4
0.90	93.5 ± 0.9	54.0 ± 3.6	54.0 ± 3.6
0.95	93.3 ± 1.1	53.8 ± 3.3	53.8 ± 3.3

Supplementary Table 2 | Validity, uniqueness, and novelty of the molecules generated during the pretraining of the chemical language models. Each experiment was repeated 10 times over 40 epochs. Every 10 epochs, 5000 molecules were sampled. The “best epoch” is defined as the epoch yielding the highest average novelty value. All percentages are reported with respect to the total number of molecules sampled.

Data	Parameter	Best epoch	Valid (%)	Unique (%)	Novel (%)
Temperature	0.7	30	97.9 ± 0.3	97.0 ± 0.3	97.0 ± 0.3
Nucleus	0.60	40	93.8 ± 0.4	93.8 ± 0.4	93.8 ± 0.4
Nucleus	0.65	40	93.5 ± 0.2	93.5 ± 0.2	93.5 ± 0.2
Nucleus	0.70	40	93.4 ± 0.5	93.4 ± 0.5	93.4 ± 0.5
Nucleus	0.75	40	93.5 ± 0.4	93.5 ± 0.4	93.5 ± 0.4
Nucleus	0.80	40	93.6 ± 0.4	93.6 ± 0.5	93.6 ± 0.5
Nucleus	0.85	40	93.7 ± 0.3	93.7 ± 0.3	93.7 ± 0.3
Nucleus	0.90	40	93.6 ± 0.4	93.6 ± 0.4	93.6 ± 0.4
Nucleus	0.95	40	93.7 ± 0.4	93.6 ± 0.4	93.6 ± 0.4

Supplementary Table 3 | K_d determination of compound 1. The signal is a transformation of the qPCR cycle time (2^{-C_t}). Two replicates are reported ($n = 2$).

Concentration (nM)	Signal (replicate 1)	Signal (replicate 2)
0	1.33×10^{-6}	1.29×10^{-6}
0	1.41×10^{-6}	1.46×10^{-6}
0	1.45×10^{-6}	1.29×10^{-6}
0.169	1.49×10^{-6}	1.36×10^{-6}
0.508	1.24×10^{-6}	1.46×10^{-6}
1.52	1.54×10^{-6}	1.43×10^{-6}
4.57	1.39×10^{-6}	1.36×10^{-6}
13.7	1.13×10^{-6}	1.11×10^{-6}
41.2	1.29×10^{-6}	1.22×10^{-6}
123	1.12×10^{-6}	1.16×10^{-6}
370	9.56×10^{-7}	8.23×10^{-7}
1110	5.12×10^{-7}	5.35×10^{-7}
3330	2.04×10^{-7}	1.78×10^{-7}
10000	6.43×10^{-7}	6.44×10^{-7}

Supplementary Table 4 | Calculated binding energies. GoldScores obtained for the docked de novo molecules and the re-docked crystal-structure ligand (1-methyl-3-naphthalen-2-yl-1*H*-pyrazolo[3,4-*d*]pyrimidin-4-amine), in the active site of PI3Kγ (PDB ID: 3ENE). RMSD = root-mean-square deviation.

Molecule	GoldScore / kJ mol^{-1}
1	-29.84
17	-34.66
18	-33.83
19	-32.83
20	-31.73
21	-31.64
22	-32.33
Redocking<br>(PDB **ID**: 3ENE, RMSD = 0.448 Å)	-33.93

Supplementary Figures

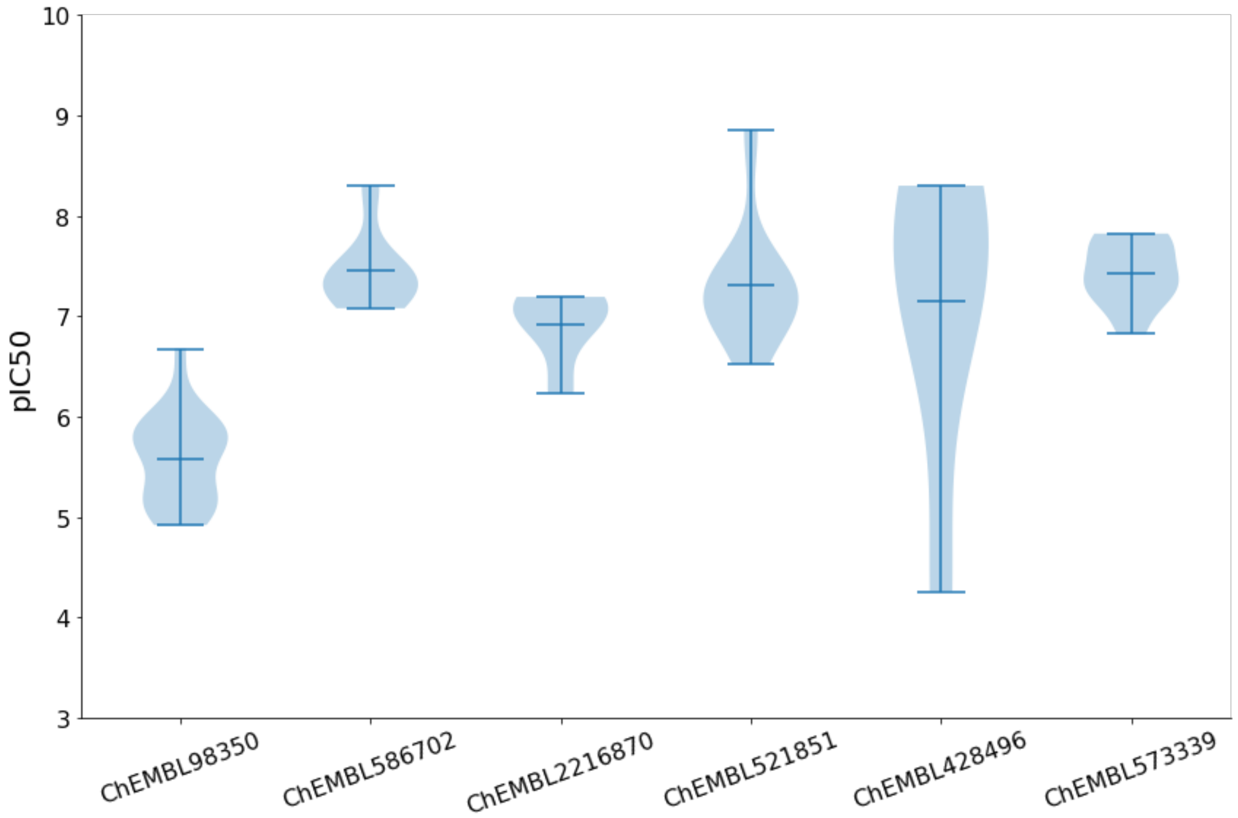

Supplementary Fig. 1 | Activity distribution range. (Left to right) Molecules with the most activity entries for PI3Kγ (Gene name: PIK3CG) in the Drug Target Commons database**.** The mean (middle line) and extremes (upper and lower lines) are shown for each violin plot. ChEMBL identifiers of the molecules are reported on the *x*-axis. Number of entries per target/violin plot: ChEMBL98350: N = 21, ChEMBL586702: N = 7, ChEMBL2216870: N = 5, ChEMBL521851: N = 9, ChEMBL428496: N = 8, ChEMBL573339: N = 9).

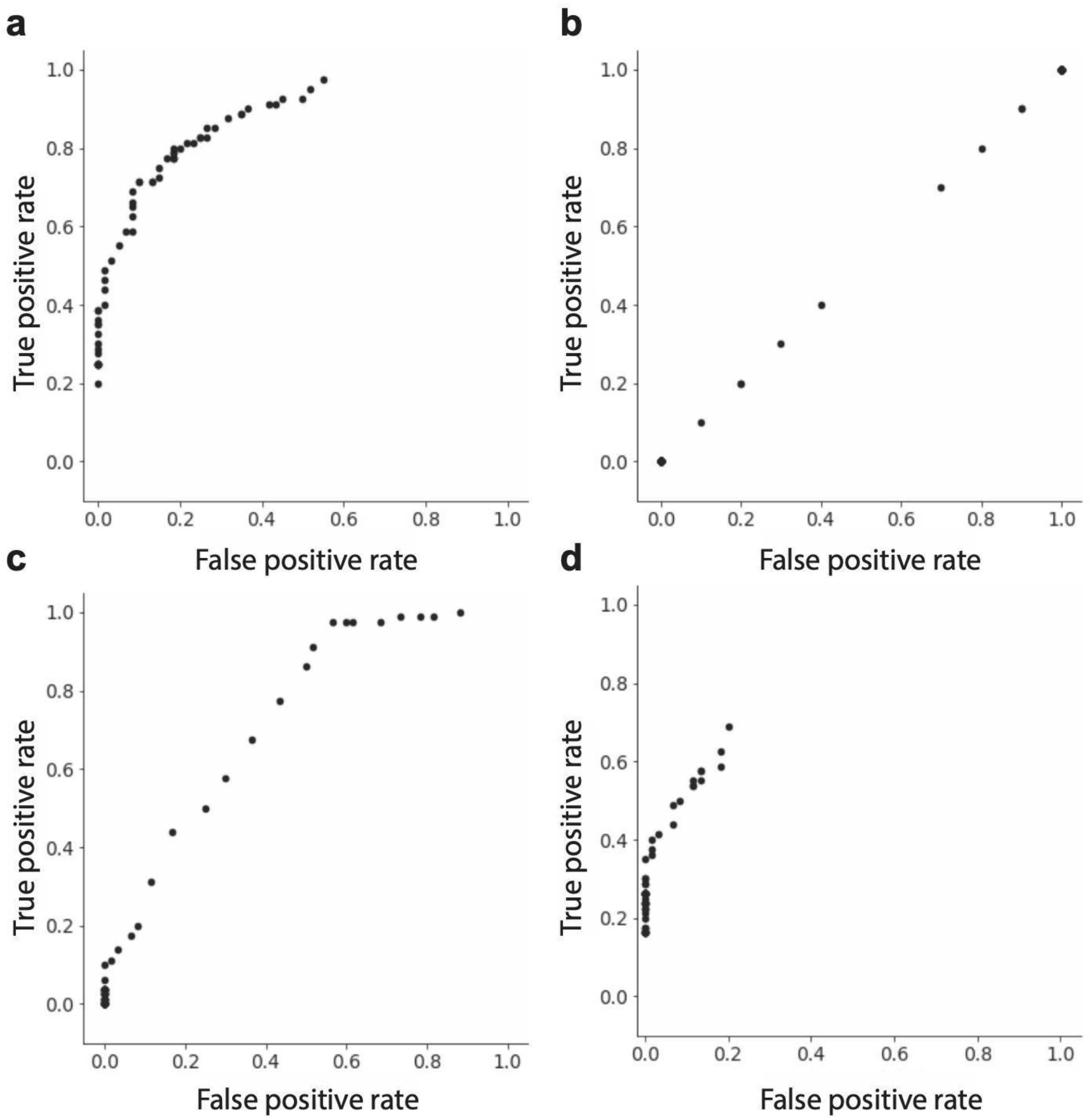

Supplementary Fig. 2 | Bioactivity prediction with the ELECTRA pretraining. The true positive rate is defined with respect to the most active class. The false positive rate refers to the nonactive molecules misclassified as "highly active". **a,** Results of the pretraining and fine-tuning of all layers. **b,** Control without pretraining and fine-tuning of all layers. **c,** Results of the pretraining and fine-tuning of the first layer while the second one was kept frozen. **d,** Results of the pretraining and fine-tuning of the second layer while the first one was kept frozen.

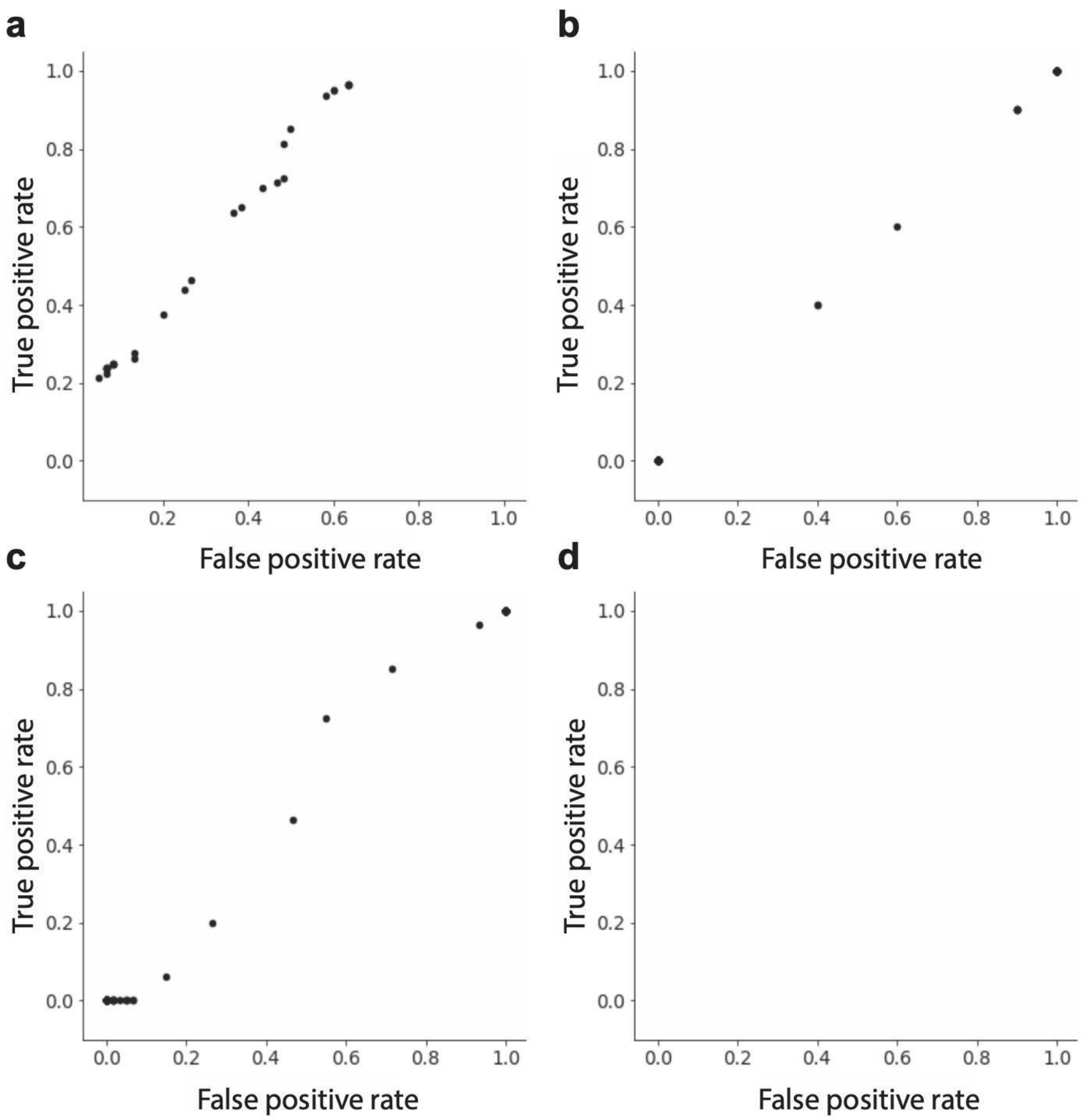

Supplementary Fig. 3 | Bioactivity prediction with the autoregressive pretraining. The true positive rate is defined with respect to the most active class. The false positive rate refers to the nonactive molecules misclassified as highly active. **a,** Results of the pretraining and fine-tuning of all layers. **b,** Control without pretraining and fine-tuning of all layers. **c,** Results of the pretraining and fine-tuning of the first layer while the second one was kept frozen. **d,** Results of the pretraining and fine-tuning of the second layer while the first one was kept frozen.

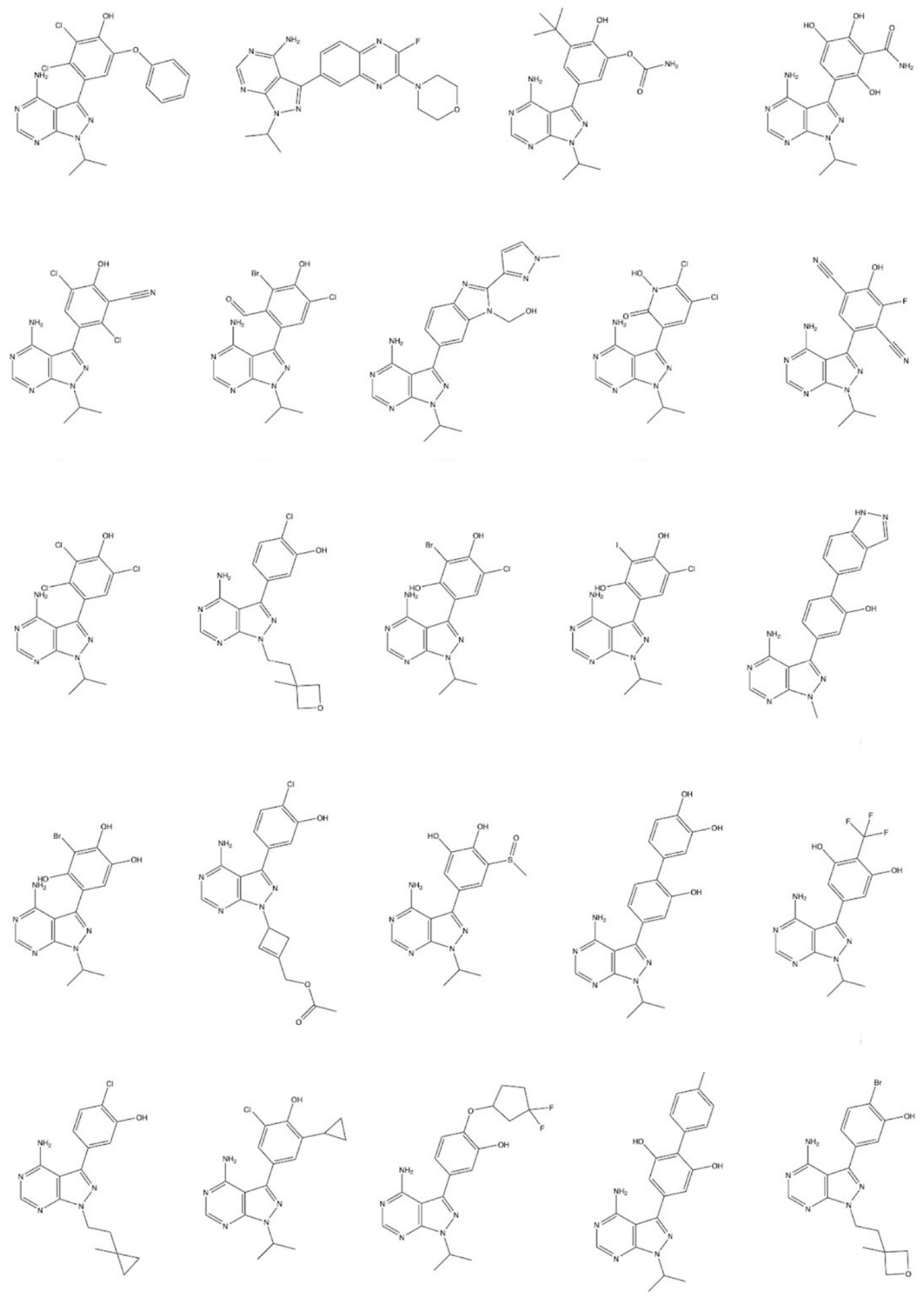

Supplementary Fig. 4 | 24 of the 47 top-ranked compounds. Each molecule obtained the top-ranked score of 99 positive votes out of 100 possible.

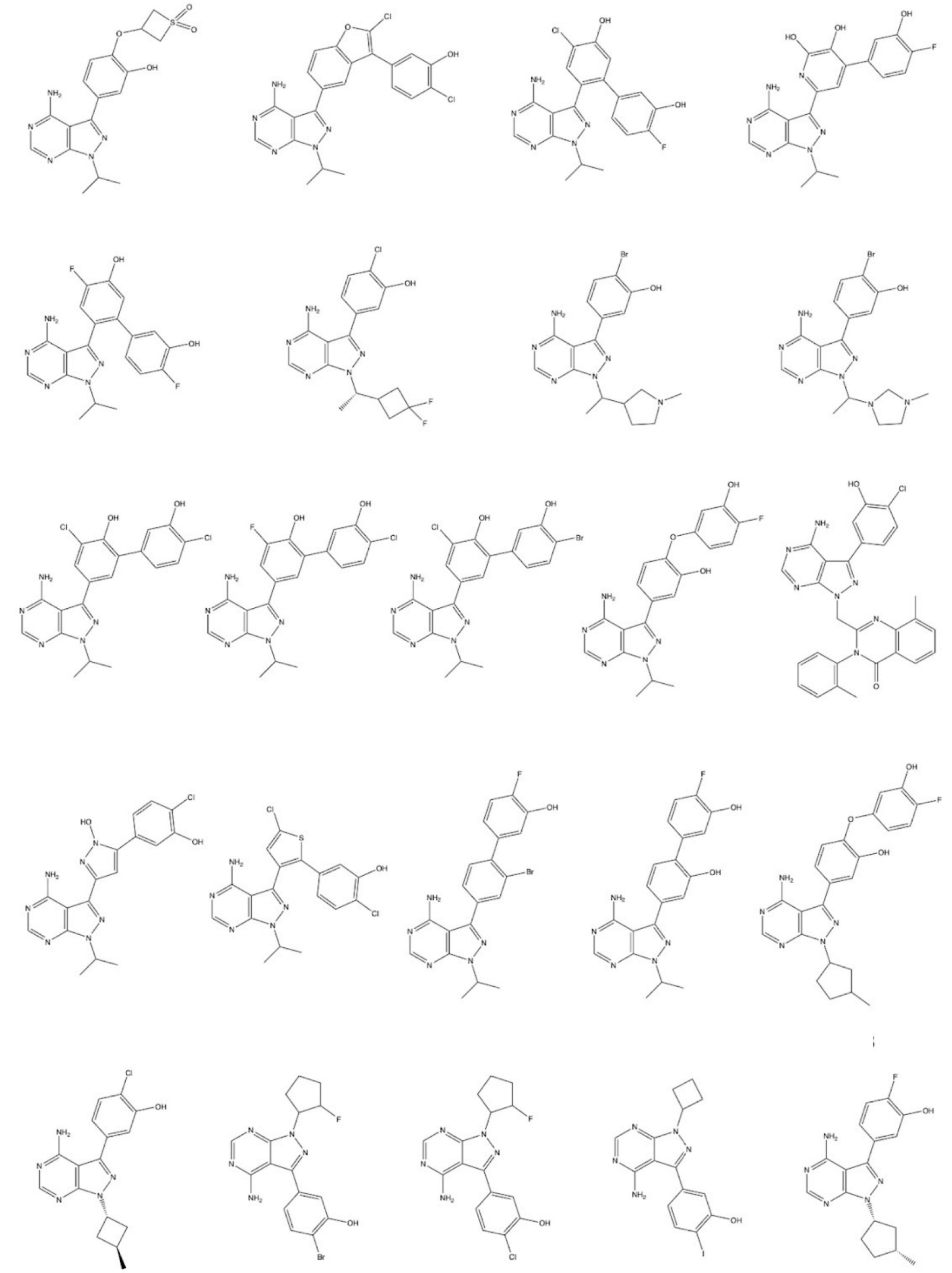

Supplementary Fig. 5 | 23 of the 47 top-ranked compounds. Each molecule obtained the top-ranked score of 99 positive votes out of 100 possible.

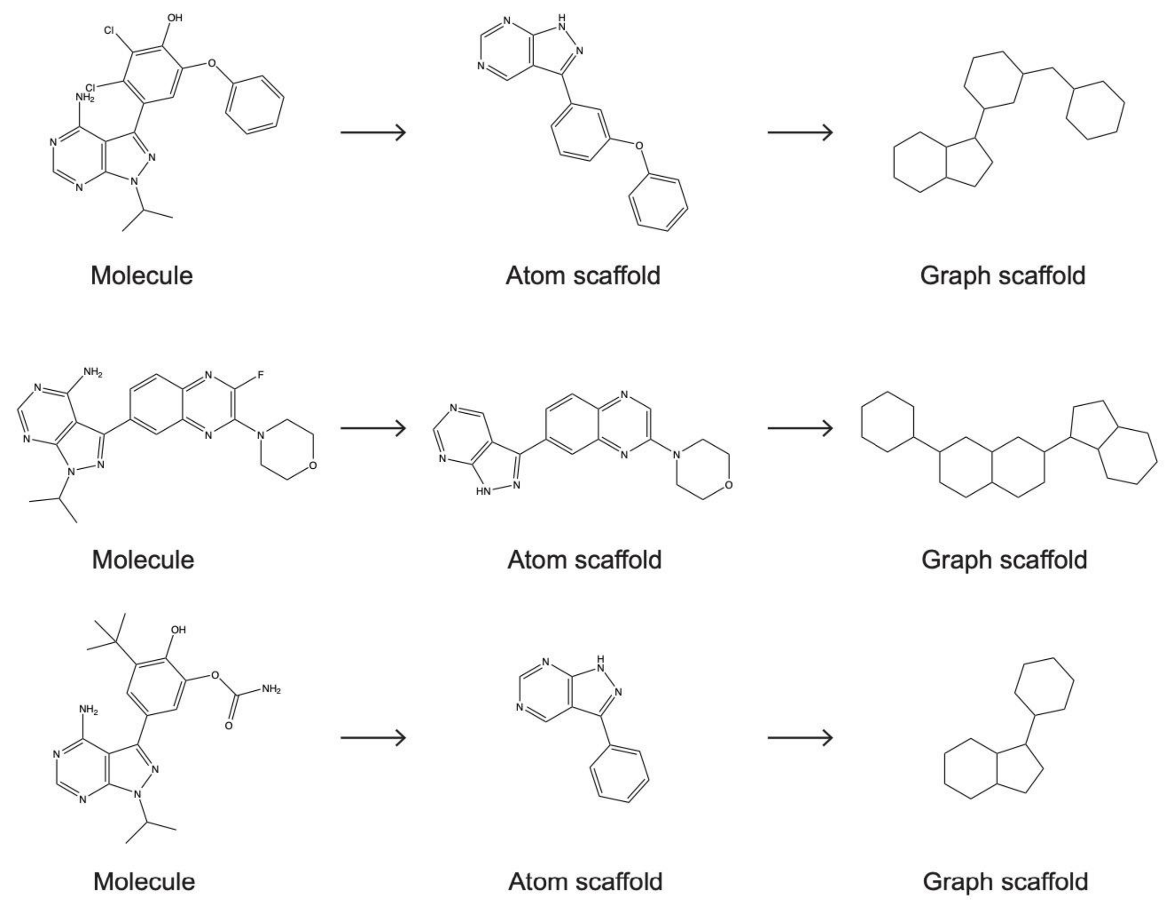

Supplementary Fig. 6 | Three examples of graph and atom scaffold decomposition from a molecule with the open-source cheminformatics software RDKit.

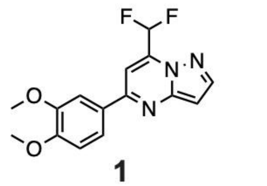

1

Equivalent pChEMBL: 6.19

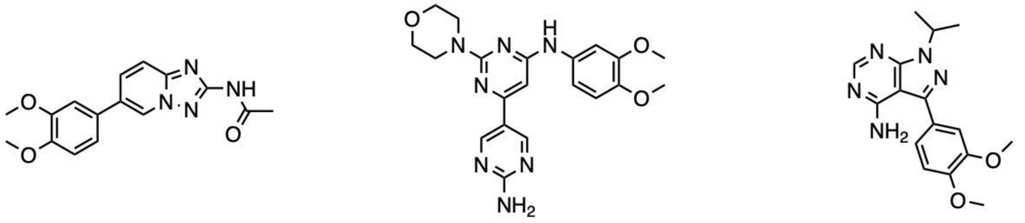

Similarity: 34%
pChEMBL: 7.4
ID: CHEMBL2064482

Similarity: 32%
pChEMBL: 6.85
ID: CHEMBL2017978

Similarity: 30%
pChEMBL: 6.7
ID: CHEMBL1242290

Supplementary Fig. 7 | Similarity analysis of molecule 1 toward known bioactive molecules. The three molecules in ChEMBL that are most similar to molecule **1**, with a reported pChEMBL ≥ 5.0 in terms of Tanimoto distance based on Morgan fingerprints (expressed as percent).

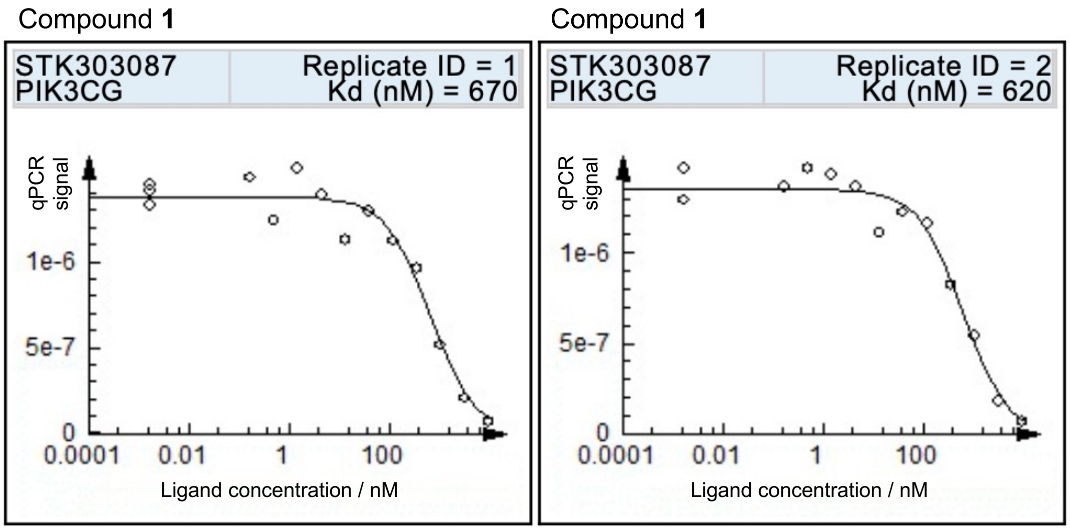

Supplementary Fig. 8 | *In vitro* characterization of compound 1. Kinase-ligand binding was determined in a competition assay (N = 2), using an immobilized ligand of PI3Kγ and quantitative polymerase chain reaction (qPCR) measuring the competing DNA-tagged PI3Kγ protein. The signal is expressed as a transformation of the qPCR cycle time (2 - cycle time).

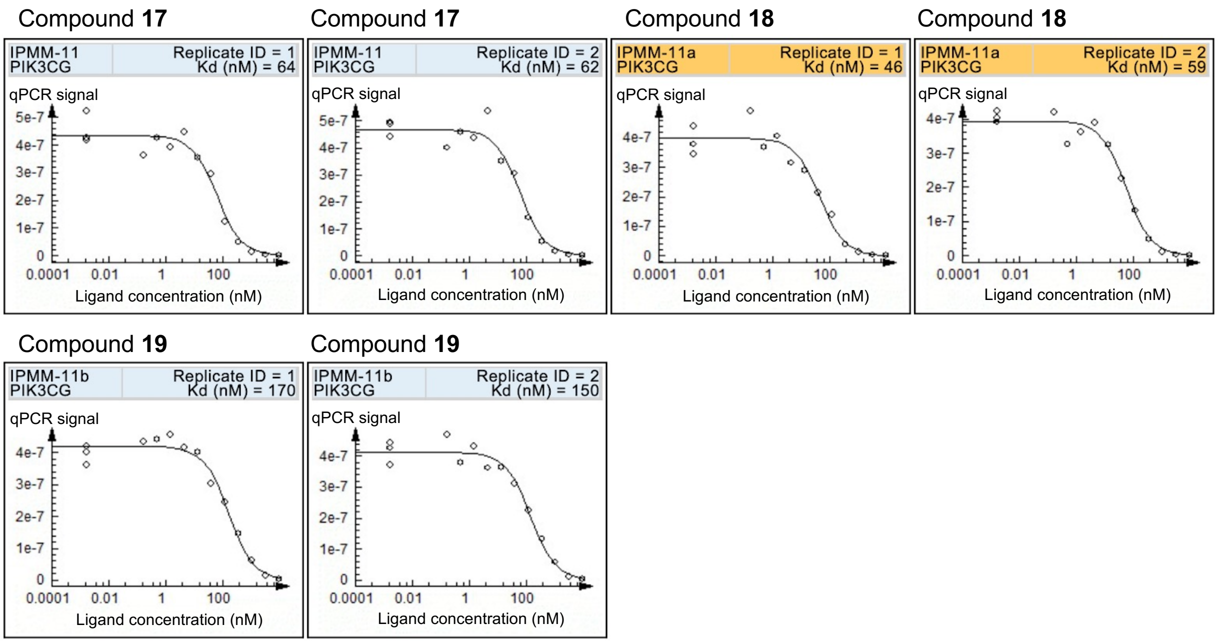

Supplementary Fig. 9 | *In vitro* characterization of compounds 17‑19.

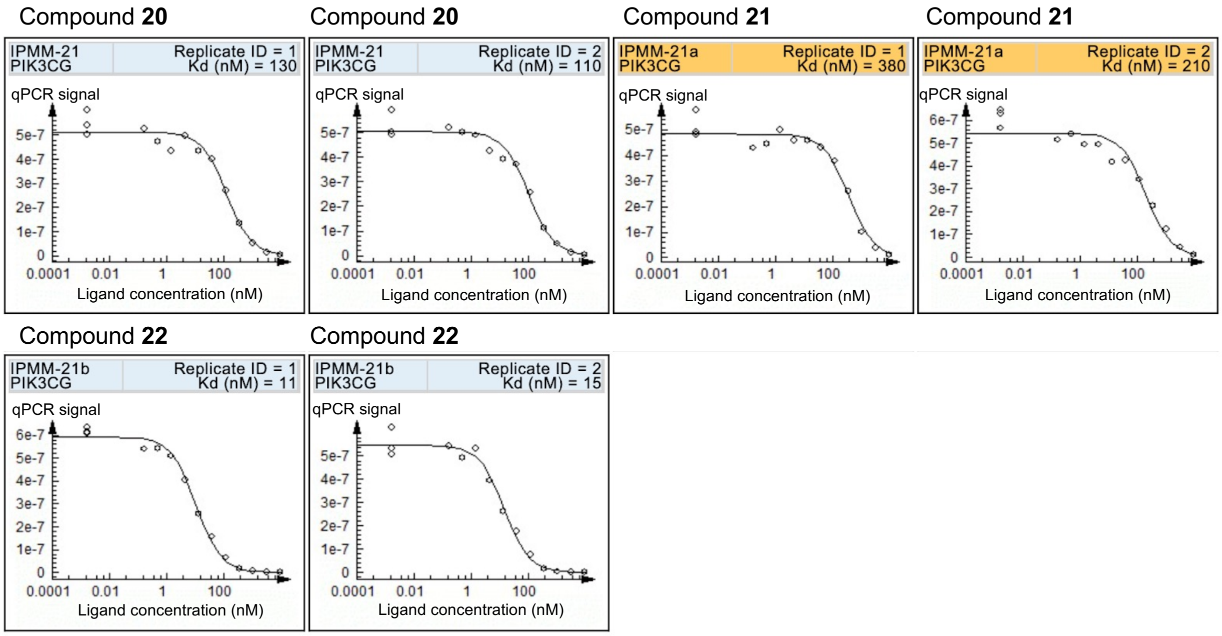

Supplementary Fig. 10 | *In vitro* characterization of compounds 20‑22.

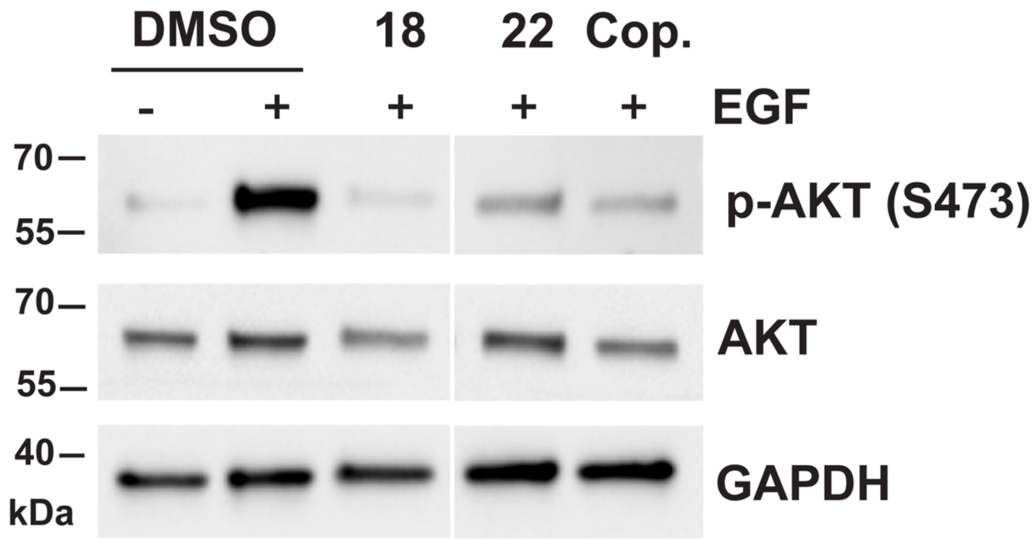

Supplementary Fig. 11 | Compounds 18 and 22 repress EGF-induced AKTS473 phosphorylation in tumor cells. Immunoblot analysis of p-AKT (S473), AKT and GAPDH (loading control) using total lysates of human HD-MB03 tumor cells stimulated with 10 ng ml^{-1} EGF for 15 min in the absence or presence of compounds **18** or **22**, or copanlisib (Cop.). Compounds and copanlisib were used at 100 nM concentration. Dimethylsulfoxide (DMSO) was used as solvent control. Biological replica of the immunoblot is shown in Fig. 6a in the main document.

Uncropped images of Western blots

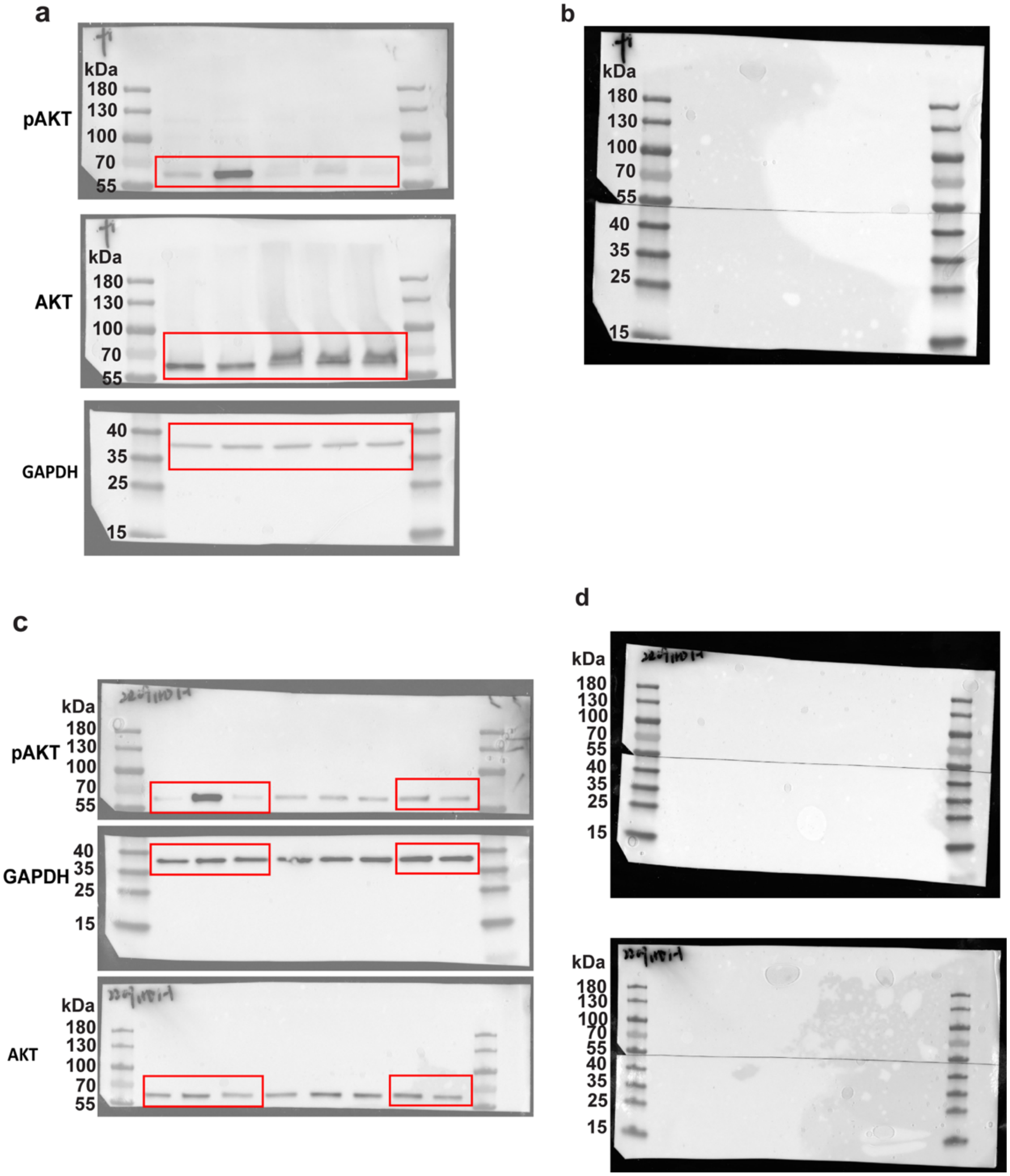

Supplementary Fig. 12 | Uncropped immunoblots and membranes of data shown in Fig 6a and Supplementary Fig. 11. a, Uncropped immunoblots of anti-phospho-AKTS473, anti-AKT and anti-GAPDH data shown in Fig. 6a. **b**, Membranes with molecular weight markers used for immunoblots shown in a. **c**, Uncropped immunoblots of anti-phospho-AKTS473, anti-AKT and anti-GAPDH data shown in Supplementary Fig. 11. **d**, Membranes with molecular weight markers used for immunoblots shown in c. Red boxes on immunoblots in a and c indicate the cropped areas shown in Fig. 6a and Supplementary Fig. 11.

Supplementary Methods

A) Overview of chemical syntheses

B) Chemical synthesis: Materials and methods

C) Chemical synthesis and analytics

C.1) Chemical synthesis: General procedures

C.2) Chemical synthesis and analytics of compounds **17**–**19**

- Synthesis of compound **17**
- Synthesis of compound **18**
- Synthesis of compound **19**

C.3) Chemical synthesis and analytics of compounds **20**–**22**

A) Overview of chemical syntheses

The PI3K inhibitors designed by the CLM are structurally defined by an aliphatic chain at position 1 and a functionalized aromatic ring at the position 3 of the pyrazolo fragment, integrated into a disubstituted pyrazolo[3,4-*d*]pyrimidin-4-amine scaffold (Fig. 1). The general synthetic strategy to achieve the exposed compounds was based on the modification in positions 1 and 3 of the commercial available starting material 4-amino-3-iodo-1H-pyrazolo[3,4-d]pyrimidine, through the corresponding alkylation process. Position 1 was alkylated with the corresponding aliphatic chain to generate the core structure intermediate (precursor 2). Precursor 2 was coupled with the corresponding aryl boronate derivates (precursor 1) at position 3, through a microwave assisted palladium cross coupling reaction (Suzuki reaction) as the key step. From the further advanced precursor intermediate the final products **17** and **20** and the corresponding analogues were obtained (Supplementary Fig. 13).

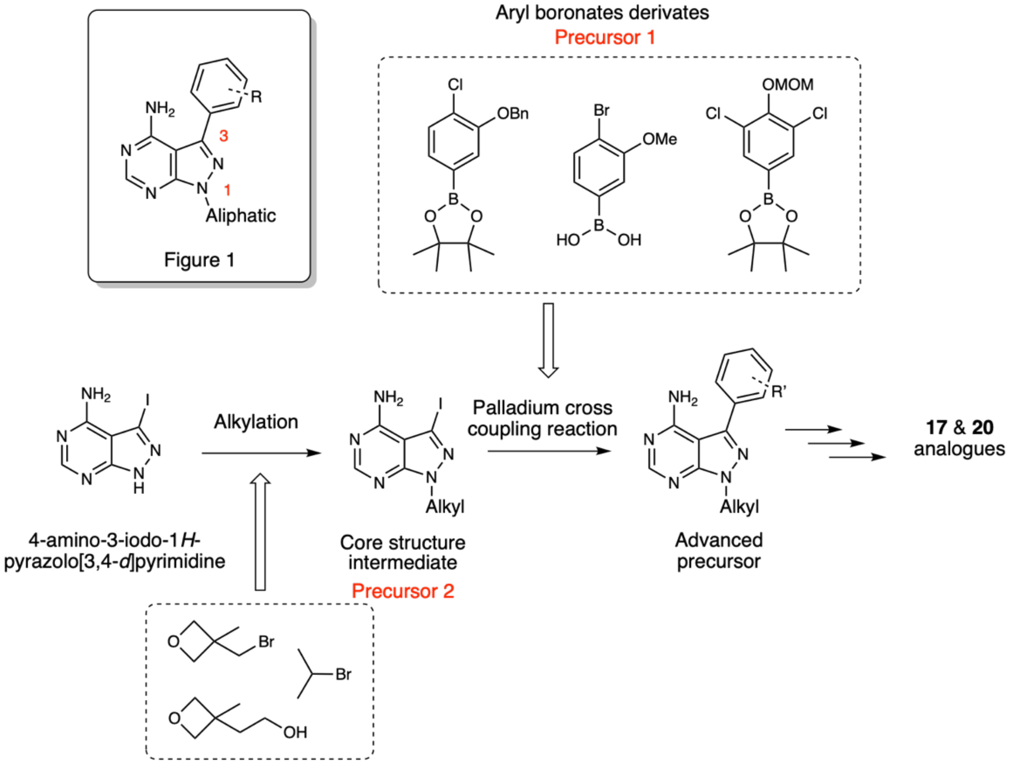

Supplementary Fig. 13 | Overview of the chemical synthesis.

The synthesis to obtain compound **17** started with the formation of the corresponding aryl boronate derivative **17b**. 2-Chloro-5-iodophenol was benzylated in mild and basic conditions with benzylbromide to yield **17a** with a 75% yield, leading to the intermediate **17b** (55% yield) after following a Miyaura-Borylation procedure. Precursor **17d** was obtained from ethyl 2-(oxetan-3-ylidene)acetate according to the procedure described by Zhang *et al.* (30% yield). The core intermediate **17e** was obtained through Mitsunobu reaction between **17d** and 4-amino-3-iodo-1*H*-pyrazolo[3,4-*d*]pyrimidine in low yields (4–8%). At that point, the precursors **17b** and **17e** were

submitted to microwave-assisted palladium cross coupling reaction conditions (GP2), providing **17f** (57% yield). Compound **17** was obtained with a 10% yield after removing the benzyl group from **17f** by hydrogenation in presence of 10% Pd/C (Supplementary Fig. 14).

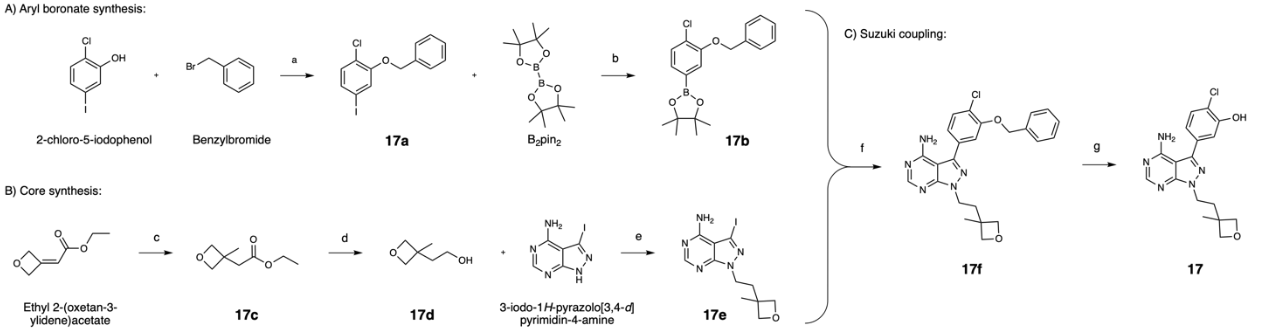

Supplementary Fig. 14 | Synthesis of compound 17. Reagents and conditions: (a) K_2CO_3, MeCN, 0°C to RT, ON; (b) AcOK, Pd(dppf)Cl_2, dry dioxane 0.3M, 80°C, overnight; (c) CuI, TMSCl, MeMgBr, dry THF, -15°C to RT; (d) $LiAlH_4$, dry tetrahydrofuran (THF), 0°C to RT, 1h; (e) diisopropyl azodicarboxylate (DIAD), PPh_3, dry THF, room temperature (RT), overnight (ON); (f) $Pd(PPh_3)_4$, Na_2CO_3, DME/H_2O, 85°C microwave (μw) 1h30; (g) H_2, 10% Pd/C, MeOH, RT, ON.

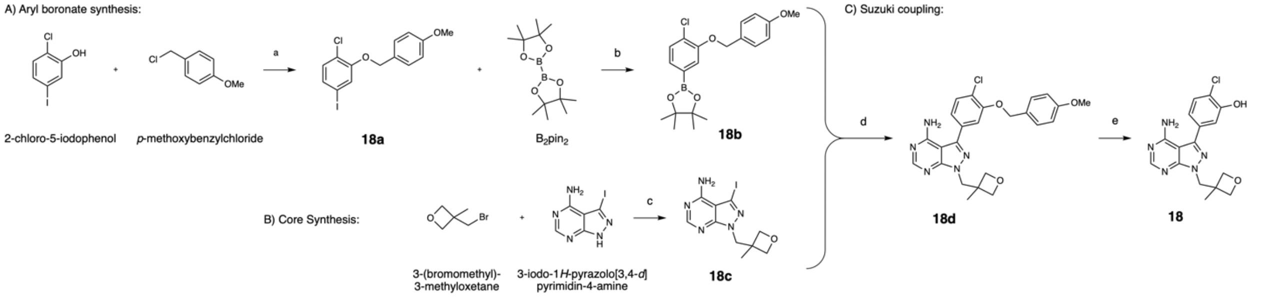

Supplementary Fig. 15 | Synthesis of compound 18. Reagents and conditions: (a) K_2CO_3, MeCN, 0°C to 60ºC, ON; (b) AcOK, Pd(dppf)Cl_2, dry dioxane 0.3M, 80°C, μw, 2h; (c) K_2CO_3, dimethylformamide (DMF), 180°C μw, 5 min; (d) $Pd(PPh_3)_4$, Na_2CO_3, DME/H_2O, 85°C μw, 1h30; (e) H_2, 10% Pd/C, MeOH, RT, ON.

The synthesis of compound **18** started with the protection of 2-chloro-5-iodophenol using a *p*-methoxybenzyl group, which resulted in the intermediate **18a** (77% yield). This step allowed the formation of **18b** (47% yield) after a Miyaura borylation[1]. The core scaffold intermediate **18c** was obtained by microwave-assisted alkylation (GP4) of 3-(bromomethyl)oxetane and 4-amino-3-iodo-1*H*-pyrazolo[3,4-*d*]pyrimidine (70% yield), irradiating at high temperature. **18b** was then coupled with **18c** through a microwave-assisted palladium cross-coupling reaction (GP2) to obtain **18d** (16% yield) which, after hydrogenation, led to the desired final compound **18** (44% yield) (Supplementary Fig. 15).

For the synthesis of **19**, the core scaffold intermediate **18c** was submitted to microwave-assisted palladium cross coupling reaction conditions (GP2) with 4-bromo-3-methoxyphenyl boronic acid to produce desired product in 34% yield (Supplementary Fig. 16).

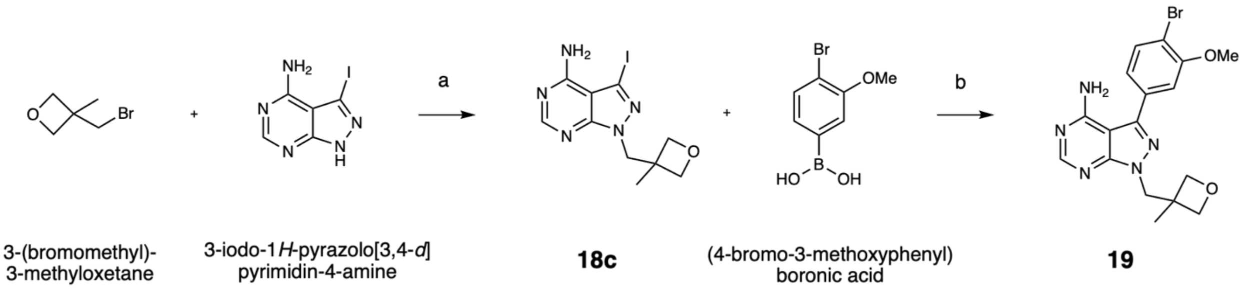

Supplementary Fig. 16 | Synthesis of compound 19. Reagents and conditions: (a) K_2CO_3, DMF, 180°C μw, 5 min; (b) $Pd(PPh_3)_4$, Na_2CO_3, DME/H_2O, 85°C μw, 1h30.

The synthetic route to obtain **20**, **21** and **22** started with the synthesis of the aryl boronate derivate **20a** (87% yield) through a MOM-protection over the -OH of 2,6-dichloro-4(4,4,5,5-tetramethyl-1,3,2-dioxaborolan-2-yl)phenol under mild basic conditions. The core scaffold intermediate **20b** was synthesized by microwave-assisted alkylation reaction (GP4) between 2-bromopropane and 4-amino-3-iodo-1*H*-pyrazolo[3,4-*d*]pyrimidine. Both precursors, the aryl boronate **20b** and the core scaffold **20c**, were coupled by microwave-assisted palladium cross coupling (GP2) to obtain **20c** (69% yield). At this point, the synthetic route branched in two pathways. The first one provided compounds **20** and **21**. After submitting **20c** to Suzuki conditions for introducing the cyclopropane ring from the corresponding boronic acid, the expected compound **20d** was obtained, together with the bi-cyclopropane-product **20e**. Flash chromatography proved insufficient to obtain pure compound. Therefore, the resulting crude of the reaction with both intermediates was deprotected under mild acid conditions to successfully afford compounds **20** and **21** with an overall yield over the last two steps of 18% and 10%, respectively. The second pathway corresponds to the synthesis of the compound **22**, after -OMOM deprotection of **20c** under acid conditions (16% yield) (Supplementary Fig. 17).

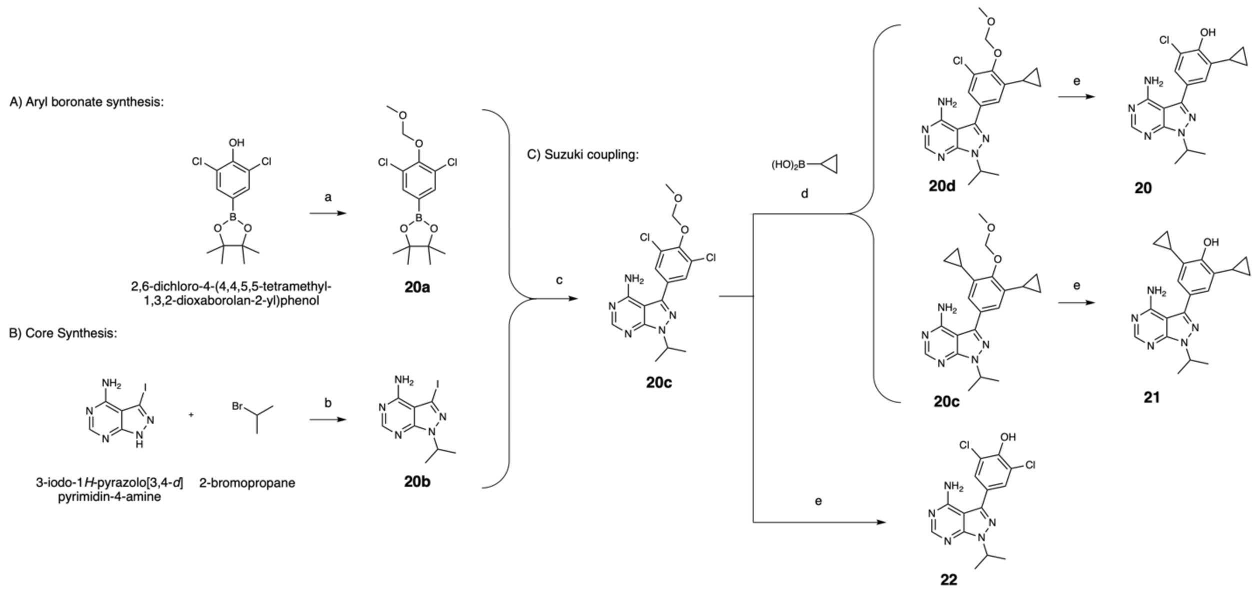

Supplementary Fig. 17 | Synthesis of 20, 21, and 22. Reagents and conditions: (a) MOMBr, K_2CO_3, MeCN, 0°C to RT, ON; (b) K_2CO_3, 180°C µw, 5 min; (c) $Pd(PPh_3)_4$, Na_2CO_3, DME/H_2O, 85°C µw 1h30; (d) $Pd(OAc)_2$, PCy_3, K_3PO_4, Toluene/H_2O, 100°C, 24h; (d) HCl, MeOH, RT, 3h.

B) Chemical synthesis: Materials and methods

Reagents, solvents, and reactions conditions

All chemicals were purchased in the highest available purity (95-99%) and were used without further purifications unless described otherwise. Anhydrous reactions were performed in oven-dried glassware (110°C), in absolute solvents, and under inert atmosphere (nitrogen or argon atmosphere). Na_2SO_4 or $MgSO_4$ were used as drying agents. Room temperature (RT) refers to 25°C. Reaction reflux conditions were obtained using DrySyn® heating blocks (Radnor, PA, USA) Equipped with a standard thermometer. Solvent evaporations were performed under reduced pressure on a Büchi rotary evaporator. Microwave (µw) reactions were carried out in a Biotage Initiator+ reactor (Uppsala, Sweden).

Purification techniques and thin layer chromatography (TLC)

All reactions were monitored by TLC using precoated silica gel aluminum plates (Macherey-Nagel, Oensingen, Switzerland) and visualized by ultraviolet (UV) light at 254 and 366 nm. Flash column chromatographies (FCC) purifications were performed using a Biotage Isolera instrument.

Nuclear magnetic resonance spectroscopy (NMR)

NMR spectra, ^1H and ^{13}C, were acquired on a Bruker AV 400 or Bruker AV 500 spectrometer (Bruker Corporation, Billerica, MA, USA). Chemical shifts (δ) are reported in parts per million (ppm) relative to tetramethylsilane (TMS) reference. Coupling constants (*J*) are reported in Hertz (Hz), and multiplicities are reported as follow: s (singlet), d (doublet), t (triplet), q (quadruplet), p (quintet), bs (broad singlet), dd (doublet of doublet), ddd (doublet of doublet of doublet), dt (doublet of triplet), m (multiplet). The analytical and spectroscopic data of each compound are in good agreement with their structures.

Mass spectrometry

High-resolution mass spectra (HRMS) were recorded on a Bruker maXis – ESI-Qq-TOF-MS (Bruker Corporation, Billerica, MA, USA). Purity of all compounds was determined by reversed phase high-performance liquid chromatography (HPLC)-MS with UV and ESI-MS detection on a Shimadzu (Kyoto, Japan) liquid chromatography–mass spectrometry (LC-MS) 2020 system with a Nucleodur C18 HTec column (150 × 3 mm, 5 µm, 110 Å; Macherey-Nagel, Düren, Germany) and a linear 5–50% acetonitrile in water (MilliQ) gradient containing 0.1% formic acid over 17 minutes with a flow rate of 0.5 ml min^{-1} at 30°C. No impurities were detected in MS or UV for all compounds submitted to biological testing.

C) Chemical Synthesis

C.1) General Procedures

General Procedure 1 (GP1) – Phenol benzylation. Commercially available 2-chloro-5-iodophenol (1 equivalent [eq]) and K_2CO_3 (3 eq) were placed in a round bottom flask (RBF), and MeCN (0.2 M) was added. The resulting mixture was stirred at RT for 5 mins before adding of benzylbromide (1.2 eq, dropwise) or the *p*-methoxybenzyl chloride (1.3 eq, dropwise). The resulting mixture was stirred at RT (benzyl bromide) or refluxed overnight (*p*-methoxybenzyl chloride). The reaction was monitored by TLC using 100% pentane as eluent. Once the benzylation process had finished in both cases, the system was diluted in diethyl ether and filtered over a pad of Celite®. The filtrate was washed three times with water, then brine, dried over Na_2SO_4 and evaporated to be used without any further purification.

General Procedure 2 (GP2) – Suzuki coupling. Pyrazolo-pyrimidin-4-amine core scaffold derivative (1 eq), boronic acid or boronate ester (1.2 eq), Na_2CO_3 (3 eq) and $Pd(PPh_3)_4$ (5 mol %) were placed in a flamed-dried microwave vial with a stirring bar. The vial was sealed and purged with three argon/vacuum cycles. Then, DME (0.15 M) was introduced using a syringe, and finally, water (3/1 v/v DME/H_2O). The mixture was degassed with a needle and argon for 5–10 min, and the reaction was irradiated in the microwave reactor at 85°C for 90min. Subsequently the system was cooled down to room temperature and the resulting system was poured into NH_4Cl saturated solution, and the crude of the reaction was extracted with ethyl acetate (three times). The organic layers were combined, washed with brine, dried over $MgSO_4$, and finally concentrated under pressure conditions to be purified by FCC.

General Procedure 3 (GP3) – Hydrogenation. The corresponding -benzyl-protected-oxetan-pyrimidin-4-amine derivate (**17f** or **18d**) (1 eq) was placed in a two-necked RBF with a stirring bar. The flask was sealed and purged with three vacuum/argon cycles. Then, dry MeOH (0.15 M) was added, and finally 10% Pd/C was added by briefly lifting the adapter. And an inert atmosphere was introduced over the system of the reaction with three vacuum/argon cycles. Subsequently, a balloon of hydrogen was connected to one of the necks and the system was purged with three vacuum/H_2 cycles. The resulting mixture was stirred at RT overnight. After that time, the crude of reaction was filtered over a pad of Celite® and the filtrate was evaporated to be purified by FCC using 100% dichloromethane (DCM) to 85/15 DCM/MeOH. Et_2O was added over the resulting residue, and a precipitate formed. Through filtration the desired compound was obtained as an off-white solid.

General Procedure 4 (GP4) – Microwave-assisted alkylation. Commercially available 4-amino-3-iodo-1*H*-pyrazolo[3,4-*d*]pyrimidine (1 eq), the corresponding bromo-alkyl derivative (1.3 eq) and K_2CO_3 (2 eq) were placed in a microwave vial. The vial was sealed and irradiated at 180°C in a microwave reactor for 5 min. The system was cooled down up to room temperature without external agent, then H_2O was added, and the crude of the reaction was extracted with

ethyl acetate. The organic layer was washed with brine, dried over $MgSO_4$, and filtered. The resulting filtrate was then evaporated and purified by flash chromatography.

General Procedure 5 (GP5) – MOM deprotection. Concentrated 37% aqueous HCl was added to a solution of the corresponding MOM-protected adduct (**20c**, **20d** or **20e**) (1 Eq) in dry MeOH (0.06 – 0.7 M) The resulting solution was stirred at RT for 3h. Subsequently, the crude was evaporated to dryness, and the resulting oily residue was resuspended in Et_2O or DCM to form a precipitate. The filtered precipitate corresponded to the desired compound.

C.2) Chemical synthesis and analytics of compounds 17–19.

- **Synthesis of compound 17**

2-(benzyloxy)-1-chloro-4-iodobenzene (17a) was synthesized according to procedure GP1 from a suspension of 2-chloro-5-iodophenol (1 eq), K_2CO_3 (3 eq) and benzyl bromide (1.2 eq, 4.72 mmol, 0.56 ml) in MeCN (0.2 M, 20 ml). The oily residue was resuspended in Et_2O, and the white precipitate was filtered to afford 1.02 g (2.97 mmol, 75% yield) of the final product as a white solid that was used in the next steps without any further purifications.

^{1}H NMR (400 MHz, $CDCl_3$) δ 7.49 – 7.43 (m, 2H), 7.43 – 7.38 (m, 2H), 7.37 – 7.30 (m, 1H), 7.28 (d, *J* = 1.9 Hz, 1H), 7.25 – 7.21 (m, 1H), 7.09 (d, *J* = 8.3 Hz, 1H), 5.12 (s, 2H). ^{13}C NMR (101 MHz, $CDCl_3$) δ 154.93, 135.98, 131.74, 130.90, 128.81, 128.36, 123.70, 127.32, 123.24, 91.36, 71.19. HRMS (*m/z*): $[M+H]^+$ calcd. for $C_{13}H_{10}ClIO$, 343.9459; found, 343.9458.

2-(3-(benzyloxy)-4-chlorophenyl)-4,4,5,5-tetramethyl-1,3,2-dioxaborolane (17b). A RBF was charged with a stirring bar, 2-(benzyloxy)-1-chloro-4-iodobenzene (**17a**) (1 eq, 1.58 mmol, 545 mg), 4,4,4',4',5,5,5',5'-octamethyl-2,2'-bi(1,3,2-dioxaborolane) (1.5 eq, 2.37 mmol, 601.84 mg), potassium acetate (3 eq, 4.74 mmol, 465.18 mg) and Pd(dppf)Cl_2 (10 mol %, 0.32 mmol, 234.14 mg). The flask was sealed, and an inert atmosphere was introduced through three argon/vacuum cycles. Then, dry dioxane was added (0.02 M, 70 ml) and the resulting system was stirred at 80ºC overnight. The reaction was monitored by TLC using 1/1 pentane/ ethyl acetate (EA) as eluent system. The resulting was cooled down to RT, then poured over saturated aqueous solution of NH_4Cl and extracted with EA three. The combined organic layers were washed with brine, and dried over Na_2SO_4. The crude was filtered, and the volume of the filtrate was removed under pressure conditions until dryness. The obtained residue was resuspended in a mixture of diethyl ether and pentane (1/1 v/v) to finally obtain the desired compound as dark brown solid (360 mg). The residue was used without any further purifications.

Ethyl 2-(3-methyloxetan-3-yl)acetate (17c). An addition-funnel was connected to a two-necked RBF. The flask was charged ethyl 2-(oxetan-ylidene) acetate (1 eq, 7.04 mmol, 1000 mg), CuI (10 mol %, 0.7 mmol, 133.3 mg) and a stirring bar, sealed with the corresponding septum in the top of the funnel and in the second neck. Finally, an inert atmosphere was introduced through

argon/vacuum cycles three times. Then, dry THF (0.6 M, 12 ml) was added with a syringe and the stirring was started to create a homogeneous suspension. Trimethylsilyl chloride (TMSCl) (2 eq, 14.08 mmol, 1.79 ml) was then added dropwise at RT. Once the addition was finished, the mixture was cooled at -15ºC using an acetone/ice bath. After 5 mins stirring at that temperature, a solution of MeMgBr 3M in Et_2O in 2 ml of dry THF was placed in the addition-funnel, using a syringe through the top septum, and was added dropwise over 90min, maintaining the temperature of the system at -15ºC. Once the addition was finished, the cold bath was retired, and the reaction was stirred at RT. The evolution of the reaction was monitored by TLC (100% pentane, using *p*-anisaldehyde or $KMnO_4$ as stain). After consumption of the starting material (at around 1h), the reaction was quenched with saturated aqueous solution of NH_4Cl. The mixture was diluted in Et_2O, the organic layer was separated, and the aqueous layer was extracted with Et_2O three times. The combined organic layers were washed with brine, then dried over $MgSO_4$, filtered, and concentrated *in vacuo* to finally be purified by FCC using pentane/EA from 100% to 1/1. 333 mg (30% yield; HRMS (ESI) $[M+Na]^+$ *m/z:* 181.0839 calculated for $C_6H_{14}O_3$, found: 181.0835) of the crude were afforded as a yellow oil. The crude product was used without further purification. HRMS (*m/z*): $[M+Na]^+$ calcd. for $C_6H_{14}O_3$, 181.0839; found, 181.0835.

2-(3-methyloxetan-3-yl)ethan-1-ol (17d). Under argon atmosphere, a solution of the previously synthesized ethyl 2-(3-methyloxetan-3-yl) acetate (**17c**) (1 eq, 3.86 mmol, 610 mg) in dry THF (0.6 M, 6.45 ml) was added dropwise to the previously cooled suspension of LAH (1 eq., 3.86 mmol, 146.51 mg) in dry THF (1 M, 3.90 ml) at 0ºC, keeping the anhydrous conditions of the system. The mixture was stirred for 1h, allowing it to slowly warm up to 10-15ºC. After the time described, the cooling bath was retired, and the crude of reaction was stirred at RT for another hour. The reaction progress was monitored by TLC (100% pentane, using *p*-anisaldehyde as stain). The resulting system was finally diluted in diethyl ether and cooled at 0ºC again. Then, 0.37 ml of water, 0.37 ml of 15% NaOH aqueous solution and 1.2 ml of water were carefully added in that order. Finally, some $MgSO_4$ was added to the crude, and the resulting system was stirred at RT for 15 min. The crude was filtered, and the filtrate was concentrated under pressure conditions. A yellow oil was obtained (332 mg, 74% yield. HRMS (*m/z*): $[M+Na]^+$ calcd. for $C_6H_{12}O_2$, 139.0731; found, 139.0730). The resulting residue was used without any further purification in the next step.

5-iodo-7-(2-(3-methyloxetan-3-yl)ethyl)-7H-pyrrolo[2,3-d]pyrimidin-4-amine (17e). A RBF was charged with a stirring bar, commercially available 5-iodo-7*H*-pyrrolo[2,3-d]pyrimidin-4-amine (1 eq, 0.99 mmol, 258.63 mg), 2-(3-methyloxetan-3-yl)ethan-1-ol (**17d**) (1.3 eq, 1.30 mmol, 150 mg) and triphenylphosphine (1.5 eq, 1.49 mmol, 389.50 mg). The flask was sealed with a septum and an inert atmosphere was introduced through three vacuum/argon cycles. Dry THF (0.18 M, 5 ml) was added using a syringe and the system was vigorously stirred to afford a homogenous suspension. DIAD (1.5 Equiv., 1.49 mmol, 301.29 mg), diluted in 2 ml of dry THF, was added dropwise over 4h at RT using a syringe. After the addition was finished, the mixture was stirred overnight at RT. The TLC eluent system used was 95/5 DCM/MeOH. At this point, the crude of

reaction was evaporated under pressure conditions, and the obtained residue was resuspended in diethyl ether. The formed precipitate, not corresponding to the desired final product, was discarded. The filtrate was concentrated *in vacuo* to finally be purified by FCC using a mixture of DCM/MeOH. The product was eluted the third at around 9% MeOH in DCM and afforded as white crystals (38.1 mg, 0.106 mmol, 8% yield).

^{1}H NMR (400 MHz, DMSO) δ 8.22 (s, 1H), 4.30 (dd, *J* = 7.9, 6.6 Hz, 2H), 4.12 (d, *J* = 5.7 Hz, 2H), 4.08 (d, *J* = 5.7 Hz, 2H), 2.13 (dd, *J* = 7.9, 6.6 Hz, 2H), 1.31 (s, 3H). HRMS *(m/z)* $[M+H]^+$ calcd. for $C_{11}H_{14}IN_5O$, 360.0315; found, 360.0316.

5-(3-(benzyloxy)-4-chlorophenyl)-7-(2-(3-methyloxetan-3-yl)ethyl)-7H-pyrrolo[2,3-d]pyrimidin-4-amine (17f) was synthesized according to procedure GP2, starting from 5-iodo-7-(2-(3-methyloxetan-3-yl)ethyl)-7*H*-pyrrolo[2,3-*d*]pyrimidin-4-amine (**17e**) (1 eq, 0.045 mmol, 16.00 mg), 2-(3-(benzyloxy)-4-chlorophenyl)-4,4,5,5-tetramethyl-1,3,2-dioxaborolane (**17b**) (1.2 eq, 0.053 mmol, 20.00 mg), Na_2CO_3 (3 eq, 0.135 mmol, 14.31 mg) and $Pd(PPh_3)_4$ (5 mol %, 0.0023 mmol, 3 mg) in DME (0.15 M, 0.3 ml) and water (0.1 ml). The crude was finally purified by FCC using DCM/MeOH as eluent system. The desired product was eluted the last at around 10% MeOH in DCM as a brown powder (11.6 mg, 0.026 mmol, 57% yield).

^{1}H NMR (400 MHz, DMSO) δ 8.27 (s, 1H), 7.60 (d, *J* = 8.1 Hz, 1H), 7.54 – 7.47 (m, 2H), 7.45 (dd, *J* = 4.8, 1.5 Hz, 2H), 7.42 (d, *J* = 7.7 Hz, 1H), 7.36 (d, *J* = 7.2 Hz, 1H), 7.26 (dd, *J* = 8.1, 1.9 Hz, 1H), 5.29 (s, 2H), 4.38 (t, *J*= 7.2 Hz, 2H), 4.15 (d, *J* = 5.7 Hz, 2H), 4.11 (d, *J* = 5.7 Hz, 2H), 2.20 (t, *J* = 7.2 Hz, 2H), 1.36 (s, 3H). HRMS (*m/z*): $[M+H]^+$ calcd. for $C_{24}H_{24}ClN_5O_2$, 450.1696; found, 450.1691.

5-(4-amino-1-(2-(3-methyloxetan-3-yl)ethyl)-1*H*-pyrazolo[3,4-*d*]pyrimidin-3-yl)-2-chlorophenol (17) was prepared according to procedure GP3, from previously synthesized 5-(3-(benzyloxy)-4-chlorophenyl)-7-(2-(3-methyloxetan-3-yl) ethyl)-7*H*-pyrrolo[2,3-*d*]pyrimidin-4-amine (**17f**) (1 eq, 0.14 mmol, 62 mg) in dry MeOH (0.15 M, 1 ml) and concentrated HCl, to finally afford the desired compound as an off-white solid (6 mg, 0.016 mmol, 11% yield).

^{1}H NMR (500 MHz, DMSO) δ 10.51 (s, 1H), 8.26 (s, 1H), 7.48 (d, *J* = 8.2 Hz, 1H), 7.27 (d, *J* = 2.0 Hz, 1H), 7.07 (dd, *J*= 8.2, 2.0 Hz, 1H), 4.37 (t, *J* = 7.1 Hz, 2H), 4.13 (d, *J* = 5.7 Hz, 2H), 4.09 (d, *J* = 5.6 Hz, 2H), 2.19 (t, *J* = 7.1 Hz, 2H), 1.35 (s, 3H). ^{13}C NMR (126 MHz, DMSO) δ 158.08, 155.81, 153.99, 153.45, 142.82, 132.64, 130.57, 120.22, 119.78, 116.13, 97.17, 81.32, 69.78, 42.68, 37.54, 22.46. HRMS (*m/z*): $[M+H]^+$ calcd. for $C_{17}H_{19}ClN_5O_2$, 360.1222; found, 360.1220.

- **Synthesis of compound 18**

1-chloro-4-iodo-2-((4-methoxybenzyl)oxy)benzene (18a) was synthesized according to procedure GP1 from a suspension of 2-chloro-5-iodophenol (1 Equiv., 8.15 mmol, 2.00 g), K_2CO_3 (3 eq., 24.45 mmol, 3.38 g) and 4-methoxybenzyl chloride (1.3 eq, 10.60 mmol, 1.1 ml) in MeCN

(0.3 M, 30 ml). The oily residue was resuspended in Et_2O, and the white precipitate was filtered to afford 2.36 g (6.30 mmol, 77% yield) of the final product as a white solid that was used in the next steps without further purification.

^{1}H NMR (400 MHz, $CDCl_3$) δ 7.40 – 7.35 (m, 2H), 7.28 (d, *J* = 1.9 Hz, 1H), 7.22 (dd, *J* = 8.2, 1.9 Hz, 1H), 7.08 (d, *J* = 8.3 Hz, 1H), 6.93 (d, *J* = 8.7 Hz, 2H), 5.04 (s, 2H), 3.82 (s, 2H). ^{13}C NMR (101 MHz, $CDCl_3$) δ 159.73, 154.96, 131.67, 130.77, 129.12, 127.97, 123.67, 123.33, 114.19, 91.36, 71.08, 55.42. HRMS (*m/z*): $[M+Na]^+$ calcd. for $C_{14}H_{12}ClINaO_2$, 396.9463; found, 396.9461.

2-(4-chloro-3-((4-methoxybenzyl)oxy)phenyl)-4,4,5,5-tetramethyl-1,3,2-dioxaborolane (18b) was obtained from previously synthesized 1-chloro-4-iodo-2-((4-methoxybenzyl)oxy)benzene (**18a**) (1 eq, 4.01 mmol, 1.50 g), 4,4,4',4',5,5,5',5'-octamethyl-2,2'-bi (1,3,2-dioxaborolane) (1.5 eq, 6.02 mmol, 1.53 g), potassium acetate (3 eq, 12.03 mmol, 1.20 g) and Pd(dppf)Cl_2 (10 mol %, 0.40 mmol, 101.83 mg), which were placed in a flamed-dried microwave vial with a stirring bar. The vial was sealed, and an inert atmosphere was introduced through three argon/vacuum cycles. Then, dry dioxane was added (0.3 M, 13 ml), and the resulting system was degassed with argon. The resulting system was irradiated at 85ºC for 2h in the microwave reactor. Once the time of the reaction finished, the resulting system was cold down up to room temperature, then poured over a saturated aqueous solution of NH_4Cl and extracted with EA three times. The combined organic layers were washed with brine, and dried over Na_2SO_4. The crude was filtered, and the filtrate was reduced under pressure conditions. The obtained residue was finally purified by FCC using pentane/DCM as eluent system. The product was eluted the second at around 60% DCM in pentane (white crystals, 720 mg). The crude was used without further purification.

3-iodo-1-((3-methyloxetan-3-yl)methyl)-1*H*-pyrazolo[3,4-*d*]pyrimidin-4-amine (18c) was prepared according to procedure GP4, starting from a suspension of 3-iodo-1*H*-pyrazolo[3,4-*d*]pyrimidin-4-amine (1 eq, 1.82 mmol, 473.2 mg), 3-(bromomethyl)-3-methyloxetane (1 eq, 1.82 mmol, 0.20 ml) and K_2CO_3 (3 eq, 5.46 mmol, 754.63 mg) in DMF (0.2 M, 10 ml). The residue was resuspended in diethyl ether to afford the desired product as a light brown powder (445 mg, 1.29 mmol, 70% yield). The crude was used without any further purification.

^{1}H NMR (400 MHz, $CDCl_3$) δ 8.38 (s, 1H), 7.93 (s, 1H), 5.55 (bs, 2H), 4.82 (d, *J* = 6.2 Hz, 2H), 4.59 (s, 2H), 4.41 (d, *J* = 6.1 Hz, 2H), 1.26 (s, 4H). HRMS (*m/z*): $[M+H]^+$ calcd. for $C_{10}H_{12}IN_5O$, 346.0161; found, 346.0159.

3-(4-chloro-3-((4-methoxybenzyl)oxy)phenyl)-1-((3-methyloxetan-3-yl)methyl)-1*H*-pyrazolo[3,4-*d*]pyrimidin-4-amine (18d) was prepared according to procedure GP2, from 3-iodo-1-((3-methyloxetan-3-yl)methyl)-1*H*-pyrazolo [3,4-*d*]pyrimidin-4-amine (**18c**) (1 eq, 0.6 mmol, 200 mg), 2-(4-chloro-3-((4-methoxybenzyl)oxy)phenyl)-4,4,5,5-tetramethyl-1,3,2-dioxaborolane (**18b**) (1.2 eq, 0.87 mmol, 325.35 mg), Na_2CO_3 (3 eq, 1.80 mmol, 190.80 mg) and

$Pd(PPh_3)_4$ (5 mol %, 0.03 mmol, 34.7 mg) in DME/H_2O (0.3 M, 2/0.7 ml). The final compound was afforded as an off-white solid (44.4 mg, 0.10 mmol, 16% yield).

^{1}H NMR (400 MHz, $CDCl_3$) δ 8.37 (s, 1H), 7.54 (d, *J* = 8.0 Hz, 1H), 7.39 (d, *J* = 8.7 Hz, 2H), 7.22 (dd, *J* = 8.0, 1.9 Hz, 1H), 6.93 (d, *J* = 8.7 Hz, 2H), 5.34 (s, 3H), 5.20 (s, 2H), 4.85 (d, *J* = 6.2 Hz, 2H), 4.60 (s, 2H), 4.42 (d, *J* = 6.1 Hz, 2H), 3.82 (s, 3H), 1.33 (s, 4H). HRMS (*m/z*): $[M+H]^+$ calcd. for $C_{24}H_{24}ClN_5O_3$, 466.1640; found, 466.1642.

5-(4-amino-1-((3-methyloxetan-3-yl)methyl)-1*H*-pyrazolo[3,4-*d*]pyrimidin-3-yl)-2-chlorophenol (18) was prepared according to procedure GP3**,** starting from previously synthesized 3-(3-(benzyloxy)-4-chlorophenyl)-1-((3-methyloxetan -3-yl)methyl)-1*H*-pyrazolo[3,4-*d*]pyrimidin-4-amine (**18d**) (1 eq., 0.10 mmol, 44.4 mg) and HCl (0.1 ml) in dry MeOH (0.15 M, 1 ml), following the work up procedure described previously to finally afford the desired compound as an off-white solid (14.6 mg, 0.042 mmol, 44% yield).

^{1}H NMR (400 MHz, TD_8F) δ 9.13 (s, 1H), 8.21 (s, 1H), 7.42 (d, *J* = 8.2 Hz, 1H), 7.24 (d, *J* = 2.0 Hz, 1H), 7.19 – 7.10 (m, 1H), 4.76 (dd, *J* = 5.9, 3.6 Hz, 2H), 4.59 (s, 2H), 4.26 (d, *J* = 5.8 Hz, 2H), 1.22 (s, 3H). ^{13}C NMR (101 MHz, THF) δ 159.66, 157.14, 154.90, 144.40, 134.70, 131.43, 121.13, 120.19, 117.54, 116.64, 116.25, 80.91, 53.67, 42.02, 22.44. HRMS (*m/z*): $[M+H]^+$ calcd. for $C_{16}H_{16}ClN_5O_2$, 346.1065; found, 346.1066.

- **Synthesis of compound 19**

3-(4-bromo-3-methoxyphenyl)-1-((3-methyloxetan-3-yl)methyl)-1*H*-pyrazolo[3,4-*d*]pyrimidin-4-amine (19). The crude was prepared according to procedure GP2, starting from 3-iodo-1-((3-methyloxetan-3-yl)methyl)-1*H*-pyrazolo[3,4-*d*] pyrimidin-4-amine (**18c**) (1 eq, 0.6 mmol, 200 mg), commercially available (4-bromo-3-methoxyphenyl) boronic acid (1.2 eq, 0.7 mmol, 160.3 mg), Na_2CO_3 (3 eq, 1.8 mmol, 190.80 mg) and $Pd(PPh_3)_4$ (5 mol %, 0.03 mmol, 34.7 mg) in DME/H_2O (0.3 M, 2/0.7 ml). The desired compound precipitated at the interface between the organic and aqueous layers. The product was afforded by filtration as a white solid (83 mg, 0.21 mmol, 34% yield).

^{1}H NMR (400 MHz, DMSO) δ 8.26 (s, 1H), 7.72 (d, *J* = 8.1 Hz, 1H), 7.31 (d, *J* = 2.0 Hz, 1H), 7.18 (dd, *J* = 8.1, 1.9 Hz, 1H), 7.02 (bs, 3H), 4.68 (d, *J* = 5.9 Hz, 2H), 4.56 (s, 2H), 4.26 (d, *J* = 5.9 Hz, 2H), 3.92 (s, 3H), 1.20 (s, 3H). ^{13}C NMR (101 MHz, DMSO) δ 158.20, 155.94, 155.66, 155.09, 143.15, 133.47, 121.52, 112.57, 111.16, 96.98, 79.30, 56.14, 52.25, 40.46, 21.76. HRMS (*m/z*): $[M+H]^+$ calcd. for $C_{17}H_{18}BrN_5O_2$, 404.0717; found, 404.0717.

C.3) Chemical synthesis and analytics of compounds 20–22

2-(3,5-dichloro-4-(methoxymethoxy)phenyl)-4,4,5,5-tetramethyl-1,3,2-dioxaborolane (20a). Commercially available 2,6-dichloro-4-(4,4,5,5-tetramethyl-1,3,2-dioxaborolan-2-yl)phenol (1 eq, 2.67 mmol, 770 mg) was dissolved in MeCN (0.3 M, 10 ml), and K_2CO_3 (3 eq, 7.99 mmol, 1105 mg) was added, and the resulting system was stirred at 0°C for 10 min. Then, MOMBr (1.2 eq., 3.20 mmol, 0.26 ml) was added dropwise. Once the addition was finished, the crude was stirred at RT overnight. The resulting mixture of the reaction was filtered over a pad of Celite®, and the filtrate was evaporated to be purified by FCC using pentane/EA (95/5 v/v). The desired product was obtained as a colorless oil (781 mg, 2.35 mmol, 87% yield).

^{1}H NMR (400 MHz, $CDCl_3$) δ 7.72 (s, 2H), 5.21 (s, 2H), 3.68 (s, 3H), 1.33 (s, 12H).^{13}C NMR (101 MHz, $CDCl_3$) δ152.07, 135.31, 129.26, 99.51, 84.59, 58.35, 24.97. HRMS (*m/z*): $[M+H]^+$*:* calcd. for $C_{14}H_{19}BCl_2O_4$, 355.0646; found, 355.0652.

3-iodo-1-isopropyl-1*H*-pyrazolo[3,4-*d*]pyrimidin-4-amine (20b) was prepared according to procedure GP4, starting from commercially available 4-amino-3-iodo-1*H*-pyrazolo[3,4-*d*]pyrimidine (1 eq, 3.83 mmol, 1000 mg), 2-bromopropane (1.3 eq, 4.98 mmol, 0.36 ml) and K_2CO_3 (3 eq, 11.99 mmol, 1.56 g). The obtained residue was resuspended in Et_2O and filtered to afford the desired compound as a light brown solid (834.2 mg, 2.75 mmol, 72% yield).

^{1}H NMR (400 MHz, $CDCl_3$) δ 8.30 (s, 1H), 8.01 (s, 1H), 6.09 (bs, 2H), 5.16 – 4.94 (m, 1H), 1.54 (d, *J* = 6.7 Hz, 6H). HRMS (*m/z*): $[M+H]^+$ calcd. for $C_8H_{10}IN_5$, 304.0054; found, 304.006.

3-(3,5-dichloro-4-(methoxymethoxy)phenyl)-1-isopropyl-1*H*-pyrazolo[3,4-*d*]pyrimidin-4-amine (20c). The crude was prepared according to procedure GP2, from 3-iodo-1-isopropyl-1*H*-pyrazolo[3,4-*d*]pyrimidin-4-amine (**20b**) (1 eq, 0.55 mmol, 166.2 mg), 2-(3,5-dichloro-4-(methoxymethoxy)phenyl)-4,4,5,5-tetramethyl-1,3,2-dioxaborolane (**20a**) (1.2 eq, 0.82 mmol, 274 mg), Na_2CO_3 (3 eq, 1.65 mmol, 174.9 mg) and $Pd(PPh_3)_4$ (5 mol%, 0.03 mmol, 31.78 mg) to be purified by FCC using pentane/EA from 100% to 2/8 pentane/EA as eluent system. The desired product eluted the last, and was obtained as a white solid (145 mg, 0.38 mmol, 69% yield).

^{1}H NMR (400 MHz, $CDCl_3$) δ 8.39 (s, 1H), 7.68 (s, 2H), 5.42 (bs, 2H), 5.26 (s, 2H), 5.19 (p, *J* = 6.7 Hz, 1H), 3.73 (s, 3H), 1.59 (d, *J* = 6.7 Hz, 6H). HRMS (*m/z*): $[M+H]^+$ calcd. for $C_{16}H_{17}Cl_2N_5O_2$, 382.0832; found, 382.0833.

3-(3-chloro-5-cyclopropyl-4-(methoxymethoxy)phenyl)-1-isopropyl-1*H*-pyrazolo[3,4-*d*]pyrimidin-4-amine (20d) and **3-(3,5-dicyclopropyl-4-(methoxymethoxy)phenyl) -1-isopropyl-1*H*-pyrazolo[3,4-*d*]pyrimidin-4-amine (20e).** Previously synthesized 3-(3,5-dichloro-4-(methoxymethoxy)phenyl)-1-isopropyl-1*H*-pyrazolo[3,4-d]pyrimidin-4-amine (**20c**) (1 eq, 0.37 mmol, 142 mg), cyclopropylboronic acid (1.7 eq, 0.63 mmol, 54.43 mg), K_3PO_4 (3.5 eq, 1.3 mmol, 278.80 mg), $Pd(OAc)_2$ (10 mol %, 0.037 mmol, 8.38 mg) and PCy_3 (20 mol %, 0.074 mmol, 20.75

mg) were placed in a RBF with a stirring bar. The flask was sealed, and an inert atmosphere was introduced with three vacuum/argon cycles. Then, dry toluene (0.15 M, 2.2 ml) and H_2O (0.7 ml) were added. The resulting mixture was degassed for 10 min with argon through a needle. The reaction was stirred at 100°C and monitored by TLC using 9/1 v/v pentane/EA as eluent system. Once the starting material was fully consumed (after 10h), the reaction was quenched with NH_4Cl saturated solution at RT, and extracted with EA. The organic layers were combined, washed with brine, dried over $MgSO_4$, and reduced under pressure conditions to be finally purified by FCC using pentane/EA (9/1 v/v) as eluent system. Two products were eluted together, and the crude was used without further purification for the next deprotection step (178 mg as a light brown amorphous solid).

4-(4-amino-1-isopropyl-1*H*-pyrazolo[3,4-d]pyrimidin-3-yl)-2-chloro-6-cyclopropylphenol (20) and **4-(4-amino-1-isopropyl-1*H*-pyrazolo[3,4-d]pyrimidin-3-yl)-2,6-dicyclopropylphenol (21)** were prepared according to procedure GP5, starting from the mixture coming from **20d** and **20e** (1 eq, 0.46 mmol, 178 mg) in 7.4 ml of dry MeOH (0.7 M) and HCl 37% aqueous solution (0.4 ml) for 3h at RT. Then, the crude of the reaction was evaporated to dryness and redissolved in DCM. A white precipitate was formed. The precipitate was filtered, providing 4-(4-amino-1-isopropyl-1*H*-pyrazolo[3,4-*d*]pyrimidin -3-yl)-2-chloro-6-cyclopropylphenol (**21**) as a white solid (14.4 mg, 0.04 mmol, 18% yield). The filtrate was evaporated, and the residue was resuspended in Et_2O. The precipitate was filtered to afford 4-(4-amino-1-isopropyl-1*H*-pyrazolo[3,4-*d*]pyrimidin -3-yl)-2,6-dicyclopropylphenol (**22**) as a light yellow solid (8 mg, 0.023 mmol, 10 % yield).

Analytics:

20: white powder; 14.4 mg, 0.04 mmol, 18%. ^{1}H NMR (400 MHz, DMSO) δ 9.50 (s, 1H), 8.21 (s, 1H), 7.42 (d, *J* = 2.1 Hz, 1H), 7.00 (d, *J* = 2.2 Hz, 1H), 5.03 (p, *J* = 6.6 Hz, 1H), 2.18 (tq, *J* = 8.7, 5.3 Hz, 1H), 1.47 (d, *J* = 6.7 Hz, 6H), 1.01 – 0.90 (m, 2H), 0.80 – 0.63 (m, 2H). HRMS (*m/z*): $[M+H]^+$ calcd. for $C_{17}H_{18}ClN_5O$, 344.1273; found, 344.1274.
21: light yellow solid; 8 mg, 0.023 mmol, 10 %. ^{1}H NMR (400 MHz, DMSO) δ 8.72 (s, 1H), 8.20 (s, 1H), 6.88 (s, 2H), 5.01 (p, *J* = 6.7 Hz, 1H), 2.16 (dq, *J* = 8.5, 4.4 Hz, 2H), 1.47 (d, *J* = 6.6 Hz, 6H), 1.08 – 0.81 (m, 4H), 0.68 – 0.56 (m, 4H). HRMS (*m/z*): $[M+H]^+$ calcd. for $C_{20}H_{23}N_5O$, 350.1975; found, 350.1977.

4-(4-amino-1-isopropyl-1*H*-pyrazolo[3,4-*d*]pyrimidin-3-yl)-2,6-dichlorophenol (22) was prepared according to procedure GP5, starting from 3-(3,5-dichloro -4-(methoxymethoxy)phenyl)-1-isopropyl-1*H*-pyrazolo[3,4-*d*]pyrimidin-4-amine (**20c**) (1 eq., 0.16 mmol, 60 mg) in 2.5 ml of dry MeOH (0.06 M) and concentrated HCl (0.1 ml) to afford the desired compound (white solid, 8.4 mg, 0.025 mmol, 16% yield).

^{1}H NMR (400 MHz, DMSO) δ 10.42 (s, 1H), 8.22 (s, 1H), 7.57 (d, *J* = 1.5 Hz, 2H), 6.98 (bs, 2H), 5.04 (p, *J* = 6.7 Hz, 1H), 1.48 (d, *J* = 6.7 Hz, 6H). ^{13}C NMR (101 MHz, DMSO) δ 158.19, 155.49, 153.35, 149.35, 141.01, 128.30, 125.91, 122.61, 97.41, 48.18, 21.75. HRMS (*m/z*): $[M+H]^+$ calcd. for $C_{14}H_{13}Cl_2N_5O$, 338.0570; found, 338.0573.

Supplementary References

1. Ishiyama,T., Murata, M. & Miyaura, N. J. *Org. Chem. 60*, 7508–7510 (1995).
2. Zhang, R. *et al*. *Angew. Chem. Int. Ed*. *58*, 18055–18060 (2019).
3. Li, X. *et al. J. Med. Chem*. *58*, 9625–9638 (2015).
