# Supplementary material for: Leveraging molecular structure and bioactivity with chemical language models for de novo drug design
Source: Nat Commun. 2023 Jan 7;14:114. doi: 10.1038/s41467-022-35692-6 (PMC9825622; doi:10.1038/s41467-022-35692-6)
Supplement: Supplementary file 3 — Reporting Summary [file 41467_2022_35692_MOESM3_ESM.pdf]

nature portfolio

Corresponding author(s): Gisbert Schneider

Last updated by author(s): 2022/11/27

Reporting Summary

Nature Portfolio wishes to improve the reproducibility of the work that we publish. This form provides structure for consistency and transparency in reporting. For further information on Nature Portfolio policies, see our Editorial Policies and the Editorial Policy Checklist.

Statistics

For all statistical analyses, confirm that the following items are present in the figure legend, table legend, main text, or Methods section.

n/a	Confirmed	
☐	☒	The exact sample size (*n*) for each experimental group/condition, given as a discrete number and unit of measurement
☐	☒	A statement on whether measurements were taken from distinct samples or whether the same sample was measured repeatedly
☐	☒	The statistical test(s) used AND whether they are one- or two-sided<br>*Only common tests should be described solely by name; describe more complex techniques in the Methods section.*
☒	☐	A description of all covariates tested
☒	☐	A description of any assumptions or corrections, such as tests of normality and adjustment for multiple comparisons
☐	☒	A full description of the statistical parameters including central tendency (e.g. means) or other basic estimates (e.g. regression coefficient) AND variation (e.g. standard deviation) or associated estimates of uncertainty (e.g. confidence intervals)
☒	☐	For null hypothesis testing, the test statistic (e.g. *F*, *t*, *r*) with confidence intervals, effect sizes, degrees of freedom and *P* value noted<br>*Give P values as exact values whenever suitable.*
☒	☐	For Bayesian analysis, information on the choice of priors and Markov chain Monte Carlo settings
☒	☐	For hierarchical and complex designs, identification of the appropriate level for tests and full reporting of outcomes
☒	☐	Estimates of effect sizes (e.g. Cohen's *d*, Pearson's *r*), indicating how they were calculated

Our web collection on statistics for biologists contains articles on many of the points above.

Software and code

Policy information about availability of computer code

Data collection	RDKit package (v. 2019.03.2, https://www.rdkit.org), Python (v. 3.6.5), Keras (v. 2.2.0, https://keras.io/), TensorFlow GPU backend (v. 1.9.0, https://www.tensorflow.org)
Data analysis	Fiji software (Schindelin, J. et al. Fiji: an open-source platform for biological-image analysis. Nat. Methods 9, 676–682 (2012)); Own code, available from GitHub: https://github.com/ETHmodlab/

For manuscripts utilizing custom algorithms or software that are central to the research but not yet described in published literature, software must be made available to editors and reviewers. We strongly encourage code deposition in a community repository (e.g. GitHub). See the Nature Portfolio guidelines for submitting code & software for further information.

Data

Policy information about availability of data

All manuscripts must include a data availability statement. This statement should provide the following information, where applicable:

- Accession codes, unique identifiers, or web links for publicly available datasets
- A description of any restrictions on data availability
- For clinical datasets or third party data, please ensure that the statement adheres to our policy

The computational framework presented in this study and the pretrained neural network weights and the training data are available as a GitHub repository at https://github.com/ETHmodlab/.

Human research participants

Policy information about studies involving human research participants and Sex and Gender in Research.

Reporting on sex and gender	N/A
Population characteristics	N/A
Recruitment	N/A
Ethics oversight	N/A

Note that full information on the approval of the study protocol must also be provided in the manuscript.

Field-specific reporting

Please select the one below that is the best fit for your research. If you are not sure, read the appropriate sections before making your selection.

[x] Life sciences   [ ] Behavioural & social sciences   [ ] Ecological, evolutionary & environmental sciences

For a reference copy of the document with all sections, see nature.com/documents/nr-reporting-summary-flat.pdf

Life sciences study design

All studies must disclose on these points even when the disclosure is negative.

Sample size	N = 4 (copanlisib) or 7 (compounds 18 or 22) combined from two independent experiments. Mean and SD are shown.
Data exclusions	No data were excluded
Replication	Two independent experiments
Randomization	N/A
Blinding	Blinding was not relevant because we performed cell-based in vitro experiments.

Reporting for specific materials, systems and methods

We require information from authors about some types of materials, experimental systems and methods used in many studies. Here, indicate whether each material, system or method listed is relevant to your study. If you are not sure if a list item applies to your research, read the appropriate section before selecting a response.

Materials & experimental systems

n/a	Involved in the study	
[ ]	[x]	Antibodies
[ ]	[x]	Eukaryotic cell lines
[x]	[ ]	Palaeontology and archaeology
[x]	[ ]	Animals and other organisms
[x]	[ ]	Clinical data
[x]	[ ]	Dual use research of concern

Methods

n/a	Involved in the study	
[x]	[ ]	ChIP-seq
[x]	[ ]	Flow cytometry
[x]	[ ]	MRI-based neuroimaging

Antibodies

Antibodies used	primary anti-AKT (#9272), anti-phospho-AKT (p-AktSer473) (#4060), anti-GAPDH antibodies (#2118); all from Cell Signaling Technology, Danavers, USA.
Validation	Used for immunoblotting. HRP-linked secondary antibodies were used to detect the primary antibodies. Chemiluminescence detection was carried out using ChemiDoc Touch Gel and Western Blot imaging system (Bio-Rad, Hercules, USA). Integrated densities of protein bands from two independent IB experiments were determined using Fiji software. Background signal was subtracted from all measurements. pAKT signal was normalized to total AKT.

Eukaryotic cell lines

Policy information about cell lines and Sex and Gender in Research

Cell line source(s)	HD-MBO3 Group 3 medulloblastoma cells. Sex: Male.
Authentication	The HD-MB03 cell line was authenticated January 2022 using single nucleotide polymorphism profiling and comparison to reference database.
Mycoplasma contamination	Cell line is regularly tested by PCR for mycoplasma contamination. Cell line was found mycoplasma negative.
Commonly misidentified lines (See ICLAC register)	No misidentified cell line was used.
